# Stress-induced epigenome changes as a risk factor in the onset of mental disorders

**DOI:** 10.3389/fpsyt.2026.1764368

**Published:** 2026-02-11

**Authors:** Liliia Shkarina, Kirill Bozov, Stalik Dzhauari, Alexandra Primak, Vsevolod Tkachuk, Yuliya Chaika, Elena Neyfeld, Maxim Karagyaur

**Affiliations:** 1 Medical Research and Education Institute, Lomonosov Moscow State University, Moscow, Russia; 2 Scientific Center for Mental Health, Moscow, Russia

**Keywords:** epigenome, epigenome changes, major depression disorder, mental disorders, schizophrenia, stress

## Abstract

The global mental health study has revealed a steady increase in the prevalence of mental disorders worldwide. This trend reflects not only the improvements in diagnostics but also the global population ageing and the intensification of negative environmental impacts that provoke the manifestation of such disorders. One of such primary external causes for mental disorders is stress, which accompanies humans throughout their lives. Stressful exposure, particularly chronic stress, can alter the expression of genes involved in the development, maturation, and functioning of the nervous system, which in turn may provoke the manifestation of mental disorders in susceptible individuals. The effects of stress can explain the increasing prevalence of mental illnesses (depression, anxiety disorders), and their aggravation with age. Stress seems to have the greatest impact during critical periods of brain development: intrauterine and early postnatal stages. The molecular mechanisms mediating the impact of stress on the expression of genes crucial for brain development and function, as well as the list of genes involved, remain poorly understood. In this review, we have attempted to summarize the known information on the influence of stress on the activity of epigenetic modifiers and the state of the epigenome, the expression of target genes, brain development, and changes in behavioral patterns. Studying such mechanisms and the genes involved opens up opportunities for diagnosing mental disorders at a new methodological level and potentially offers new precision approaches to their therapeutic correction at the epigenomic level.

## 1 Introduction

The incidence of psychiatric disorders (schizophrenia, major depression disorder (MDD), autism spectrum disorders (ASD), anxiety disorders, post-traumatic stress disorder (PTSD), among others) is steadily increasing worldwide. Thus, from 1990 to 2019, the number of people suffering from mental disorders increased by 48%, reaching 970 million. The incidence of schizophrenia rose by 66%, from 14.2 million to 23.6 million people, while the number of patients with MDD increased by 63%, from 171 million to 280 million. This trend establishes them as one of the most pressing challenges in modern healthcare (1). The rise in the incidence of psychiatric disorders is attributed to both improved diagnostics and the growing impact of adverse environmental factors, such as pollution, poor nutrition, and social stress.

The etiology of mental disorders is multifactorial. The classical model of psychiatric disease pathogenesis posits that genetic predisposition is realized into mental illness primarily under the influence of adverse environmental factors, particularly intense or chronic stress. This is supported by twin studies showing that genetic predisposition alone is often insufficient for disease onset, and that environmental influences play a substantial role in disease induction. This raises the question: how can environmental exposures, such as stress, alter the biological program of an organism, leading to the manifestation of mental disorders?

Epigenetic DNA modifications and subsequent altered gene expression may serve as this crucial link between environmental influence and disease pathogenesis, as indicated by current scientific evidence. Epigenetic mechanisms act as a dynamic molecular interface that “reads” signals from the environment and adapts the cellular transcriptome to external conditions, influencing cell function and phenotype. Similarly, environmentally induced changes in gene expression represent a key molecular bridge linking environmental exposures (particularly stress) to persistent alterations in brain function and behavior.

Arise of a mental disorder is a complex process of interplays between the environment, genetics and epigenetics, where each of the components can influence the others (2–4). External environmental factors can alter activity of the epigenetic mechanisms and provoke genomic mutations; genetic mutations can cause epigenetic aberrations and changes in the environment (becoming a cause of new stressors); disruptions in epigenetic regulation, in turn, can induce environmental changes and induce new genetic mutations (5). Thus, a closed loop of mutual influence is formed, where each system component can be both a cause and a consequence of changes in others. These components can also synergize, increasing the likelihood of disease manifestation (2–4).

Among environmental factors, stress holds a special place (6–8). Every aspect of human life - from diet and emotional experiences to trauma and aging - activates signaling pathways and modulates activity of epigenetic modifiers via changes in hormone and neurotransmitter levels (9), ultimately leaving its mark on the individual’s epigenetic landscape (6, 10, 11).

Given that the most critical time for brain development are embryonic and early postnatal periods, severe or chronic stress can have the most damaging effect during these stages of brain morphogenesis. There are many examples that severe stress in pregnant mothers can significantly increase the onset risk for mental disorders in their children (12–15). Understanding these mechanisms and genes involved gives us an opportunity to prevent the devastating effect of stress on mental health, to prevent and treat psychiatric disorders with greater precision.

The interesting fact is that different people possess different threshold of stress resistance, and this may partly explain differences in individual susceptibility to the onset and progression of mental illnesses (16, 17). Individual genetic and/or epigenetic traits may lay the basis for personal differences in sensitivity to stress. This is confirmed by data on the use of epigenome-targeting pharmaceuticals for the treatment of mental illness. Although they demonstrate some therapeutic activity and alleviate the disease course in some cases, their limited specificity and efficacy remain a problem. The reason for poor efficacy, apparently, also lies in the individual susceptibility embedded precisely at the genetic and epigenetic levels. Insufficient specificity of these drugs is based on the complexity of epigenomic regulations and our poor understanding of these mechanisms. The only way to overcome these limitations is to identify the molecular and genetic basis of pathogenesis of mental disorders, the mechanisms of their heritability and inherited susceptibility that in future will allow developing safe and effective personalized therapeutic approaches (18). Given the exponential growth of experimental data in this field, their periodic updating and re-evaluation is required.

Therefore, the aim of this study is to systematize the accumulated experimental data to assess the potential influence of emotional stress on the epigenetic regulation of neurogenesis genes, particularly in the context of a genetic predisposition to mental disorders.

## 2 Methodology of the study

Here, we used the PubMed, ScienceDirect, and eLIBRARY.RU databases, as well as the Google search engine, to find papers on the stress-induced epigenome changes that may provoke the onset of mental disorders. The keywords used are “epigenome”, “epigenetic modifications”, “stress-induced changes”, “genetic variants”, “emotional stress”, “mental disorders”, “schizophrenia”, “major depression disorder”, “post-traumatic stress disorder”, “autism-spectrum disorders”, “heredity”, “NR3C1”, “glucocorticoid”, “NR3C2”, “FKBP5”, “MeCP2”, “HDAC”, “DNMT” and combinations thereof. As a result of the search, more than 2000 of manuscripts were detected and analyzed, of which 160 were used to write this review. They included 78 experimental articles and 80 reviews. The median publication date of the sources used was 2012, with the earliest being 2000 and the latest being 2025.

## 3 Mechanisms of epigenetic regulation of gene activity

Epigenetic modifications are chemical changes to DNA or DNA-associated proteins (histones) that alter gene activity without changing primary DNA sequence. They can be heritable, reversible or irreversible. These modifications include DNA methylation, covalent histone modifications, and all kinds of regulation mediated by non-coding RNAs. These mechanisms dynamically regulate DNA accessibility for transcription factors and other regulatory molecules, shaping the functional profile of the genome (8). Disruptions in epigenetic patterns, induced by endogenous or exogenous factors, can be strongly associated with a predisposition to and can be a direct cause of mental and neurodegenerative disorders (19–22).

The most studied DNA modification is the methylation of the fifth carbon of cytosine (5mC), catalyzed by DNA methyltransferases (DNMTs) using S-adenosylmethionine as a methyl group donor (19, 23). The primary targets for methylation are CpG sites (a cytosine followed by a guanine), although CpG islands (regions with high CpG density) typically remain unmethylated due to the presence of the activating mark H3K4me3, which impedes DNMT binding (19).

However, methylation also occurs in non-CpG contexts (e.g., CpA, CpT, CpC), particularly in embryonic tissues, stem cells, and neurons, where it accounts for up to 53% of total methylcytosine and plays a crucial role in brain development regulation (24, 25). DNA methylation is not restricted to promoters; it also occurs in gene bodies, intergenic regions, and so-called intragenic promoters (26).

The effect of DNA methylation on gene expression is highly context-dependent, determined by the location of the mark, cell type, chromatin state, and the properties of the proteins that recognize it (27). Promoter methylation typically suppresses transcription by hindering the binding of activators (e.g., CREB, MYC, AP-1) and recruiting repressors (28), whereas, gene body methylation can either suppress (29) or enhance expression (30, 31).

DNA demethylation occurs passively (during replication) or actively - through the oxidation of 5-methylcytosine to 5-hydroxymethylcytosine (5hmC), 5-formylcytosine (5fC), or 5-carboxylcytosine (5caC) by ten-eleven translocation (TET) enzymes, followed by excision repair (10, 19, 32). These demethylation intermediates also actively participate in transcriptional regulation. The level of 5hmC is particularly high in postmitotic neurons, where it is associated with active transcription and counteracts aberrant methylation (9, 33, 34).

The dynamics of methylation depend on the cell replication rate in a tissue. In actively proliferating tissues, inactive genes passively lose methyl marks, with the demethylation rate typically higher in late-replicating regions. In brain tissue, however, lowly expressed genes are more methylated than highly expressed ones. Researchers have suggested that “localization of active genes in early replication time zones allows efficient maintenance of methylation where it is apparently required (i.e. in active gene bodies), while reducing the cost of methylation maintenance at the inactive portion of the genome where a precise methylation level is presumably not essential” (35).

Beyond cytosine methylation, adenine methylation (6mA) has been described (36) that also may regulate the processes of neural development, regeneration and degeneration, although its functional role remains poorly understood.

In addition to DNA modifications, histone proteins, which form the nucleosome core, play an essential role in DNA packaging and gene accessibility (37). Histones undergo diverse covalent modifications mostly on their N- and C-terminal tails, including acetylation, methylation, phosphorylation, ubiquitination, and others (38). These modifications are catalyzed by writer enzymes (e.g., histone acetyltransferases HATs, histone methyltransferases KMTs) and removed by eraser enzymes (histone deacetylases HDACs, histone demethylases KDMs) (10, 21, 39).

Histone acetylation, catalyzed by HATs (e.g., p300/CBP), neutralizes the positive charge of histone tails, weakening their interaction with DNA, facilitating access for transcription factors (e.g., CREB), and promoting the transcription of genes involved in neurogenesis (e.g., neuropeptides, neurotrophic factors, guidance molecules). Deacetylation of acetylated histones by HDACs leads to chromatin condensation and transcriptional repression. Both HAT and HDAC families are expressed in the brain and are critical for neuroplasticity.

Histone methylation, catalyzed by KMTs, is an another kind of modification that can either activate or repress transcription depending on the modified amino acid residue and the degree of methylation. Histone-modifying enzymes often function within multi-subunit protein complexes. For instance, the repressive Polycomb Repressive Complex 2 (PRC2) catalyzes trimethylation of histone H3 at lysine 27 (H3K27me3) via its EZH2 methyltransferase subunit. The RING1 subunit within the Polycomb Repressive Complex 1 (PRC1) monoubiquitinates histone H2A (H2AK119ub). These actions ultimately lead to chromatin compaction and gene silencing (40).

Interestingly, different histone tails can simultaneously carry both activating and repressing marks, even within a single nucleosome. Genes within such “bivalent chromatin” can transit between active and silent states depending on conditions. The combination of various histone modifications forms a “histone code” that can either facilitate or impede gene expression (10, 41, 42).

Given the crucial role of histones in gene regulation, it is evident that an imbalance in histone modifications (e.g., induced by stress), particularly during critical stages of nervous system development, disrupts brain formation and functioning, laying the basis for neurodegenerative and mental disorders (21, 43–45).

The epigenetic regulatory system involves not only enzymes that add or remove marks but also reader proteins that recognize these marks and recruit transcriptional machinery. For example, heterochromatin protein 1 (HP1) binds the H3K9me3 mark and recruits HDAC4/5 for chromatin condensation (10, 46). Methyl-CpG-binding domain proteins (e.g., MeCP2) interact with co-repressors (HDAC3, NCoR complex) to suppress transcription (28). MeCP2 can also bind repressive histone marks (H3K27me3) and interact with TCF20/PHF14, RNA polymerase II, and other factors, acting as a multifunctional transcriptional regulator.

Epigenetic enzymes frequently operate within complexes (e.g., HDAC1/2 with Sin3A, NuRD, CoREST; HDAC3 with NCoR/SMRT), ensuring their specific targeting and coordinated action on genomic loci (10, 47). Disruption of this balance by stress, nutritional deficits, or mutations can cause pathologies (9), such as neurodegenerative and mental diseases. Understanding the mechanisms of targeted recruitment of epigenetic modifiers is a key aspect of developing directed therapeutic strategies for currently incurable pathologies (18), including mental disorders.

## 4 Stress and its mechanisms of influence on epigenetic modification systems

Stress is a complex physiological response of an organism to adverse environmental factors. In the context of mental disorders, emotional stress is of particular importance, acting as a key factor that initiates a cascade of molecular events leading to persistent changes in brain function.

**Figure 1:**
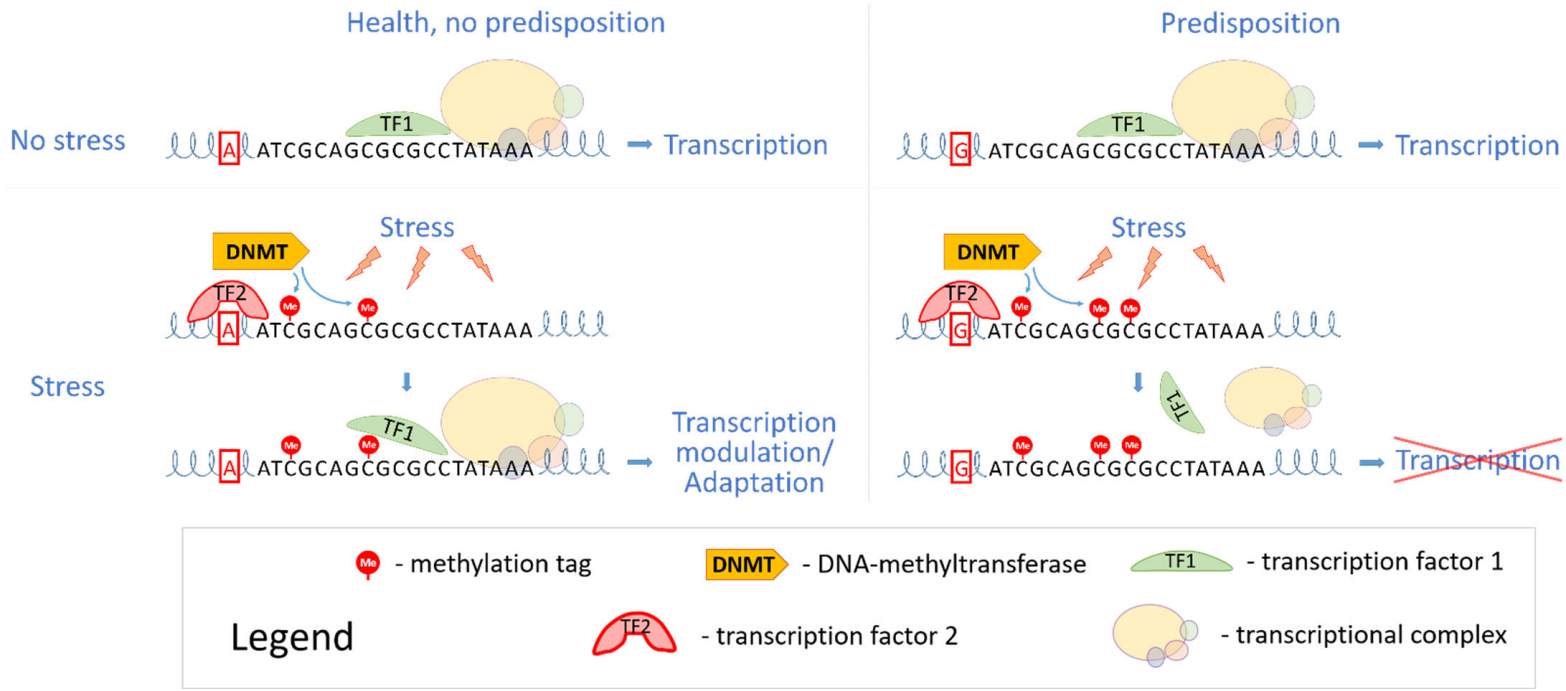
A diagram of the effect of stress on the epigenetic profile and transcriptional activity of genes against the background of individual genetic variants.

According to current understanding, the fundamental scheme of stress-induced mental disorder pathogenesis is as follows: Stress alters the levels of hormones and neurotransmitters in the central nervous system (CNS). These molecules, binding to their receptors, cause changes in the expression or activity of epigenetic modifiers. By adding or removing epigenetic marks on DNA and chromatin, epigenetic modifiers alter gene expression, which in turn changes cell behavior and phenotype (9). Certain genomic variants can affect the rate and efficiency of activation/inactivation of epigenetic modifiers, influence their expression levels and binding affinity to target DNA regions, thereby accelerating or amplifying the manifestation of functional deviations at the cellular or organismal level (**Figure 1**).

The effect of stress is largely determined by the timing and duration of exposure, with the most significant and long-term consequences arising during critical periods of CNS development (7, 21, 44), particularly the prenatal and early postnatal periods.

Prenatal stress leads to increased levels of maternal glucocorticoids, which can cross the placental barrier and re-program the fetal stress response system to prepare the offspring for a potentially stressful environment (48–50). Studies in mice have shown that prenatal exposure has a greater impact on the brain epigenome than postnatal exposure, and positive prenatal experience can mitigate the negative effects of postnatal stressors (7, 21, 51).

The prenatal stage is the most critical during brain morphogenesis, as it requires high coordination between the processes of proliferation, migration, differentiation, and apoptosis of neural progenitors, as well as neurite growth and the wiring of the brain’s compartments (52). These processes are coordinated by a wide range of molecules, including growth factors and guidance molecules, neurotransmitters, proteases, matrix proteins, etc. Alterations in the epigenetic regulation of relevant genes modify expression or production of these molecules thereby establishing a predisposition to severe neurological or mental disorders.

**Figure 2:**
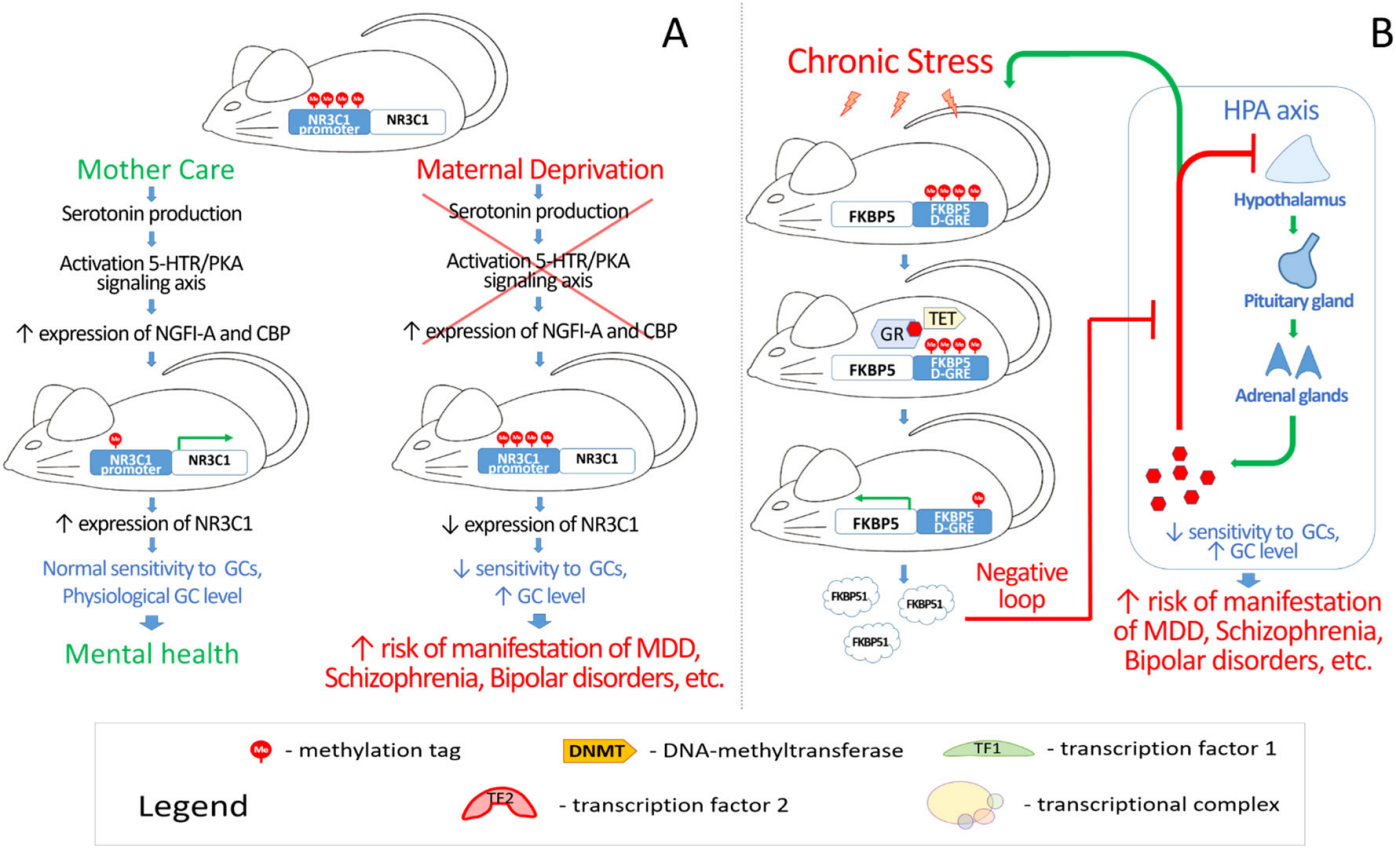
Several mechanisms have been established to explain the influence of stress on mental health: **(A)** a scheme reflecting the influence of maternal deprivation on mental health of the murine/rat pups; **(B)** a diagram demonstrating the mechanism of stress-induced disruption of the HPA axis. D-GRE - distal intronic GRE, GC - glucocorticoids, GR - glucocorticoid receptor.

The early postnatal period is characterized by active synaptogenesis and maturation of the limbic system. Therefore, severe and chronic stress during this period can substantially disrupt the formation and functioning of neural circuits. Models of maternal deprivation in rodents and studies on humans who experienced abuse demonstrate persistent, epigenetically mediated downregulation of the glucocorticoid receptor (*NR3C1*) gene, leading to lifelong hypothalamic-pituitary-adrenal (HPA) axis hyperreactivity and an increased risk of psychopathology (**Figures 2A, B**).

Epigenetic changes induced by chronic stress in adulthood are typically more reversible and less disruptive compared to the profound changes that takes place during early critical developmental periods (7, 21, 51, 53).

Before reviewing the genes, implicated in neural tissue development, and susceptible to epigenetic modifications under stress, we will outline the main mechanisms of stress-mediated activation of epigenetic regulators. Among the main mediators between emotional environmental factors and epigenomic changes is the HPA axis (54). The effect of the HPA axis on the cells in a living organism is mediated predominantly by glucocorticoid and mineralocorticoid steroid hormones. The primary function of glucocorticoid hormone reception is carried out by glucocorticoid (GR) and mineralocorticoid (MR) receptors, which, upon translocation to the nucleus, act as transcription factors by binding to glucocorticoid-responsive elements (GREs) in target genes (55, 56). The specific response of a cell to glucocorticoids is determined by chromatin accessibility, which in turn depends on cell type and differentiation state (57, 58).

A key example of the influence of HPA axis dysregulation on the development of mental disorders is provided by the study of Michael Meaney and Moshe Szyf which demonstrated that maternal care in rats modulates DNA methylation and histone acetylation in the promoter of the glucocorticoid receptor gene *NR3C1*, increasing its expression in the offspring’s hippocampus. Adequate maternal care also increases serotonin-mediated activation of cAMP-dependent protein kinase (PKA), elevating the expression of the immediate early gene *EGR1* (encoding NGFI-A protein) with subsequent recruitment of the histone acetyltransferase CREB-binding protein (CBP). By reducing *NR3C1* promoter methylation and increasing its expression, this enhances glucocorticoid sensitivity, stabilizes HPA axis function, and thereby promotes greater stress resilience in the offspring (8, 59, 60).

Individuals with mental disorders often exhibit reduced glucocorticoid receptor expression in various brain regions (56). Hypermethylation of promoter regions and reduced expression of the *NR3C1* gene is considered a cause of HPA axis hyperactivation that may trigger the onset of a variety of psychiatric disorders: MDD, schizophrenia, bipolar, anxiety and mood disorders, etc. (61–63). Meanwhile, HPA axis reduced activity is usually observed in PTSD (62, 64, 65).

Thus, in multiple studies a strong correlation between early-life stress and increased methylation of the *NR3C1* promoter regions was demonstrated (66, 67). It, in turn, conduces to a lesser nuclear translocation of GR in hippocampus cells and impairs social behavior in rodents (68). Normalization of *NR3C1* expression and GR nuclear translocation reduces HPA axis activation and restores active social interaction of mice and rats (68, 69).

The effects of glucocorticoids on neural development have been studied *in vitro* using CNS cell lines, ranging from primary neuronal cultures to organoids and assembloids (70). It allowed confirming the ability of glucocorticoids to activate epigenetic mechanisms and cause epigenetic modifications in GRE regions of GR target genes, changing their sensitivity and leading to glucocorticoid resistance.

*In vitro* studies established that the most pronounced effects of glucocorticoids occur during proliferation and neuronal differentiation stages, while effects at other stages are expressed to a lesser extent. These results confirm the critical importance of glucocorticoid levels for brain morphogenesis, especially at the stage of neural stem cells proliferation, migration and differentiation (70–72).

Bose et al. found global DNA hypomethylation in the cortex of 3-day-old mouse pups and in neural stem cells (NSCs) derived from rat embryonic cortex treated with dexamethasone *in vitro.* Dexamethasone, a synthetic glucocortocoide, was used to imitate stress, when excessive amounts of glucocortocoides are observed in blood serum.

. The effect correlated with increased TET3 and decreased DNMT3a expression in proliferating NSCs, and persisted in daughter cells never directly exposed to dexamethasone. This indicates that even “transient exposure to excess glucocorticoids have dramatic and long-lasting effects on the epigenome of NSCs and specifically point to a critical Tet3-mediated dysregulation of Dnmt3a and Dkk1” that are crucial for proper forebrain development (71). However, the observed effect disappears over time (73), it may cause epigenetic changes within the promoters of other genes. In the study by Weder et al. (74), genome-wide methylation analysis of saliva from maltreated traumatized children revealed depression-associated methylation in the bodies of *ID3*, *GRIN1*, and *TPPP* genes, involved, respectively in the stress response, neural plasticity, and neural circuitry formation.

Ensink et al., investigating methylome-wide associations in traumatized youth (8–18 years) with PTSD, identified differential methylation of CpG sites in nine statistically significant genes compared to non-traumatized healthy youth: *CRHBP*, *TNXB*, *PM20D1*, *DUSP22*, *GDF7*, *SLC1A4*, *KLHL35*, *ZNF714* and *OLFM3*. *CRHBP*, the gene of corticotrophin-releasing hormone (CHR) binding protein plays a modulatory role in CRH signaling within the brain and its hypermethylation downregulates the HPA axis. Hypermethylation of at least three genes (*PM20D1*, *TNXB*, and *OLFM3*) turned out to correlate with the alterations (volume reduction) in brain structures involved in PTSD, particularly anterior hippocampus (75).

It is a curious fact that what appears to be the same trigger (stress) can, in some instances, lead to the activation of the HPA axis, causing MDD, schizophrenia, or bipolar disorder, while in other cases, it can suppress the HPA axis, triggering the onset of PTSD. No definitive explanation for this phenomenon exists in current literature. It is hypothesized that this may be explained by the properties of the stressor (its type, intensity, duration, and timing), by the involvement of additional signaling cascades that modulate the effects of stress (12, 56–58, 76–81) and by individual characteristics of the affected person. These characteristics include the excitability and robustness of the nervous system, the state of the brain’s cellular epigenome, the profile of expressed genes, and the presence of a unique pattern of genetic variants.

Discussing the HPA axis, one cannot overlook one of its key regulators - the co-chaperone protein FKBP51 (gene *FKBP5*), which plays a critically important role in the negative feedback loop that restrains HPA overactivation and terminates the stress response. FKBP51 impedes the binding of glucocorticoid hormones to the GR receptor and hinders its nuclear translocation. Meanwhile, GR regulates the transcription of the *FKBP5* gene through its distal intronic GREs, forming an ultra-short feedback loop of the glucocorticoid response. Prolonged administration of corticosterone to mice reduces methylation in the enhancer region of intron 1 of *FKBP5*, increases levels of the FKBP51 protein, limiting the nuclear translocation of GR. These processes suppress negative control and increase the activity of the HPA axis (**Figure 2B**) (82, 83). Thus, aberrant FKBP51 activity can disrupt HPA axis negative feedback and lead to glucocorticoid “insensitivity” (77), which may underlie mental abnormalities.

FKBP51 can also influence DNA methylation by displacing FKBP52 from its complex with cyclin-dependent kinase 5 (CDK5), reducing its ability to activate DNMT1 (84). That is, the expression of FKBP51, which depends on GR through an ultra-short feedback loop, is able to influence DNA methylation.

Therefore, persistent epigenetic changes may form the basis for increased stress susceptibility, which, under chronic and intensive stress may launch the pathogenesis of a mental disorder (72, 81). Despite progress in studying stress-induced epigenetic changes, the mechanisms of *NR3C1* gene epigenetic control, HPA axis misregulation, and subsequent events that lead to glucocorticoid resistance, remain to be fully elucidated (56). It is also important to remember the complexity of epigenomic regulation, involving parallel activity of alternative signaling cascades and microRNAs (85, 86).

The activity of epigenetic modifiers is regulated not only by the HPA axis and glucocorticoids but also by a wide array of other signals. These include hormones, neurotransmitters (e.g., glutamate, serotonin), growth factors, and cytokines, which realize there activity via signaling cascades such as cAMP/PKA, PLC/PKC, PI3K/AKT, ERK/MAPK, p38 MAPK, NFκB, etc. For instance, activation of PLC or cAMP-dependent activation of PKA by growth factors, cytokines, or morphogens elevates cytoplasmic Ca²^+^ levels. This, in turn, activates Ca²^+^/calmodulin-dependent kinase IV (CamKIV), which then activates the histone acetyltransferase CBP. CBP is recruited in a complex with CREB to regulate sensitive genes, and neurotrophic factors (e.g., BDNF) among them (87).

Increased cytoplasmic Ca2+ also activates the Ca2+-dependent neuronal isoform of nitric oxide synthase (nNOS), raising intracellular nitric oxide (NO) levels and leading to S-nitrosylation of nuclear factors, including HDACs, impairing their activity and enhancing gene expression.

Signaling cascades activating ERK trigger phosphorylation of Mitogen- and Stress-activated Kinase 1 (MSK1) and the transcription factor Elk-1. Activated MSK1 phosphorylates serine-10 residues in histone H3 tails (H3S10p) in promoter regions of various genes, activating their expression. Phosphorylated Elk-1 binds to Serum Responsive Elements (SREs) in promoters and recruits the histone acetyltransferase p300, leading to histone acetylation and activation of expression of responsive genes (80, 88).

The transcriptional modulator Methyl CpG binding protein 2 (MeCP2) plays a critically important role in regulating gene activity under physiological conditions and upon stress. Initially thought to be a transcriptional repressor, modern data suggest its role as a transcriptional modulator capable of both suppressing and activating gene expression. Bin Akhtar et al. elucidated the mechanism leading to activation of genes interacting with MeCP2 (89). Thus, phosphorylation of MeCP2 at serine-421 by calcium-dependent protein kinase II (CamK II) disrupts MeCP2 binding to methylated promoter regions of target genes (e.g., *BDNF*) and leads to gene activation (90). Interestingly, reduced expression or function of MeCP2 itself can mimic the effects of stress. Cosentino et al. confirmed that reduced *MECP2* gene expression is associated with childhood stress exposure, particularly in girls, and subsequent anxiety/depression symptoms in women (91). Abellán-Álvaro et al. also showed that early weaning stress and MeCP2 deficiency in mice led to anxiety-like and depression-like behavior in adults. Reduced MeCP2 activity alone was sufficient to mimic the effects of early maternal deprivation (92) that was consistent with the findings of Cosentino et al. (91), highlighting the role of MeCP2 as a crucial molecule in stress response.

Levenson et al. established a direct link between neurotransmitter signaling and epigenetic regulation in the hippocampus. They showed that N-methyl-D-aspartate (NMDA) receptor activation triggers histone H3 acetylation in the CA1 region via the signaling pathways involving the activation of PKC/PKA and Ras/ERK (93), that triggers gene expression. Moreover, the efficacy of this process can be enhanced by administering HDAC inhibitors like trichostatin A or sodium butyrate.

The aforementioned serotonergic signaling is also involved in regulating gene expression. Increased serotonin production in the hippocampus of rat pups with good maternal care activates the transcription factor NGFI-A, increases histone acetylation of *GAD1* promoter, redounding *GAD1* expression, and decreases DNMT1 expression (94). Altogether, these mechanisms provide physiological brain development and prevent the onset of psychopathologies.

A particularly intriguing and promising avenue of research involves the potential to modulate the activity of epigenetic modifiers through dietary interventions or gut microbiota correction. Metabolic processes supply essential cofactors (such as acetyl-CoA and S-adenosylmethionine) for epigenetic editors, thereby directly influencing their activity and gene expression (95). This approach opens possibilities for the correction and prevention of a range of diseases caused by epigenetic dysregulation, including cancers, metabolic and psychiatric disorders (96–98). While this field is relatively novel and underexplored, a growing body of evidence demonstrates the potential of dietary and gut microbiota interventions to alleviate symptoms and the course of mental diseases such as depression, autism, etc. (99–101). Further research is required to elucidate the precise molecular mechanisms underlying this phenomenon that is crucial for enhancing the efficacy and expanding the applications of this relatively simple and side-effect-free therapeutic strategy.

The parallel, and often cooperative or competitive, interplay of the entire variety of signaling cascades and mechanisms underpins the complexity and dynamics of observed epigenetic modifications, brain morphology, and mental activity. The mechanisms by which various signaling cascades control the activity of epigenetic modifiers are discussed in detail in previously published reviews (80, 102, 103).

However, which molecules critical for brain development exhibit stress-induced alterations in their expression? Here are just a few of them: RELN, BDNF, ESR1, AVP, CRF, and POMC. All these molecules play a pivotal role in proliferation, migration, and/or differentiation of neural progenitors, the formation and maturation of neural connections, as well as the apoptosis of redundant cells and the pruning of non-functional synapses. Dysregulation of their activity (due to untimely, insufficient, or excessive production) disrupts brain development, in total, and may lay the pathogenetic basis for mental disorders.

Thus, it was shown that the level of methylation of the *RELN* gene, which encodes reelin - a protein involved in neuronal migration and cognitive brain functions - can change throughout life (104) as well as in some mental illnesses. Analysis of postmortem brains from schizophrenia patients revealed increased methylation of the *RELN* promoter region (105, 106), potentially mediated by the recruitment of DNMTs and HDACs involving MeCP2, which plays a key role in transmitting the stress signal.

The activity of the *BDNF* gene, encoding the most important regulator of the development, maintenance and functioning of nervous tissue, the brain neurotrophic factor (BDNF), can also change as a result of stress. BDNF supports neuron proliferation, differentiation, maturation, and survival, and plays an important role in neural plasticity. Zheng et al. established a correlation between depressive-like and anxiety-like behavior in mouse offspring caused by prenatal stress and increased methylation and decreased acetylation in promoter regions of the *BDNF* gene in the hippocampus. This coincided with increased expression of DNMT1, HDAC1, and HDAC2 compared to non-stressed offspring (107). Martinowich et al. found that CpG methylation in the *BDNF* promoter region, followed by stress-mediated recruitment of MeCP2, leads to a drastic reduction in BDNF expression through the involvement of HDAC1 (108). Interestingly, neuronal depolarization stimulates the dissociation of the repressive MeCP2-HDAC1-mSin3A complex from the *BDNF* promoter, followed by the recruitment of CREB, which activates BDNF expression (108). The effects of stress on BDNF expression can vary depending on sex, genetic background, and brain region. Kundakovic et al. showed that neonatal stress caused by maternal deprivation increased BDNF expression in the cortical area of both male and female Balb/cJ murine pups, but decreased BDNF expression in the hippocampus of female C57BL/6J mice (109).

Emerging evidence points to a critical interplay between estrogen and neurotrophic signaling. Together with the findings that estrogen receptor (ESR) genes are susceptible to epigenetic modifications under emotional stress this indicates that ESR genes may play a role in the pathogenesis of mental disorders (110–112). Champagne et al. demonstrated that female mice receiving high maternal care exhibited increased expression of the *ESR1* gene (encoding estrogen receptor ERα) compared to the offspring experiencing low maternal care. The effect was due to reduced methylation of the *ESR1* promoter and was transmitted to the next generation of female mice (113, 114). This correlated with data from humans: Fiacco et al. found increased methylation of the *ESR1* promoter region in blood cells of women who experienced severe early-life adversity (115).

Multiple studies support the fact of mutual action between estrogen and neurotrophin systems. For instance, estrogen enhances BDNF production (116), while the estrogen receptor ERα is involved in activation of the TrkB (117) and modulation of expression of tropomyosin receptor kinases TrkA and TrkB that serve as receptors for NGF and BDNF, respectively (116, 118). Thus, estrogen can regulate the processes underlying brain morphogenesis and neural plasticity. The significance of estrogen and estrogen receptors for brain development and functioning was previously described in detail (119–121).

A critically important fact is that stress exposure activates genes that sustain the stress response, thereby closing a pathological stress-induced signaling cascade. Murgatroyd et al. found that early-life stress led to persistent HPA axis hyperactivity due to hypomethylation of regulatory CpG sites in the arginine vasopressin (*AVP*) and corticotropin-releasing factor (*CRF*) genes. This, in turn, reduced MeCP2 affinity to these genes, impaired the recruitment of HDAC2 and DNMT1, led to increased CRF and AVP expression, and enhanced corticosterone production (122–124).

Similar data were obtained regarding the role of the transcriptional modulator MeCP2 in the expression of the *POMC* (pro-opiomelanocortin) gene, which is cleaved to yield adrenocorticotropic hormone (ACTH), activating adrenal function. In an experiment by Wu et al., the early-life stress modeled by daily pup separation from the mother caused increasing the *POMC* gene expression due to reduced DNA methylation in its regulatory region, an effect that persisted for up to one year (Y. 125).

Stress-induced epigenetic modification can also affect regulatory regions of other genes: the serotonin transporter *SERT* (*SLC6A4*) (126, 127), the oxytocin receptor (*OXTR*) (128), and many others (85, 129, 130). Studies on the role of gene methylation in the pathogenesis of mental disorders have yielded varied results and conclusions (131–133). This lack of consensus is a critical consideration for the development of reliable diagnostic and prognostic biomarkers.

The use of high-throughput methods (e.g., EWAS - epigenome-wide association studies) has significantly expanded the list of genes and regulatory regions confirmed to be susceptible to epigenetic modifications under the influence of the emotional environment. Epigenetic modifications can spread over large genomic regions, affecting several genes simultaneously (69). The emotional environment (negative, in particular) can have extensive, ambiguous, and multidirectional effects on the epigenome, due to the interference of its effects with hormonal signals, genomic background, and other regulatory mechanisms, including non-coding RNAs (78, 85), which often complicates the unambiguous interpretation of the results.

An important fact is that epigenetic modifications can be transmitted across generations, thereby establishing the basis for behavioral and psychiatric pathologies in offspring (49, 50, 134). It is suggested that the increased incidence of schizophrenia during times of food starvation may be partly explained by metabolic changes and, consequently, epigenomic alterations that are then inherited by offspring (40). The fundamental processes underlying this phenomenon - the so-called transgenerational epigenetic inheritance - require further studies (135–138).

The totality of the presented data allows us to state that untimely or inadequate activity of genes involved in brain development and function, arising from stress exposures, especially during early prenatal periods, can disrupt brain formation and lay the basis for mental and cognitive diseases.

## 5 Interaction of genetic variants and emotional environment in forming predisposition to mental illness

As mentioned above, the development of the brain is a finely tuned process that requires the coordinated expression of a wide range of genes in many types of cells. It is logical to assume that mutations in the genes responsible for signaling, recording, erasing, or reading epigenetic tags can disrupt this coordination, leading to desynchronization or even stopping these processes, affecting brain development and laying the material background for mental and cognitive disorders (139). Such mutations/genomic variants do not necessarily lead to knockout of the gene, but rather alter its expression level or the activity of its protein product, for example, by changing its inducibility, sensitivity to external signals, or ability to inactivate.

A known example of such a point mutation are genetic variants like rs701848-C and rs1085308044-C in the *PTEN* gene (140, 141), which impair phosphatase activity. This prevents the timely inactivation of signaling cascades triggering cell division and leading to tumor development.

In the context of mental disorders, it is even more complex, as for many hereditary psychiatric pathologies, universal genetic deviations characteristic of every patient cannot be found. The conviction grows stronger that various psychiatric disorders share overlapping sets of genetic predispositions. For instance, 109 loci influencing two or more disorders were discovered, with 83% related to schizophrenia, 72% to bipolar disorder, and 48% to major depressive disorder. Individual polymorphisms, such as rs7193263 in the *RBFOX1* gene or rs8084351 in the *DCC* gene, can be associated with seven and eight mental disorders, respectively (142–144).

**Figure 3:**
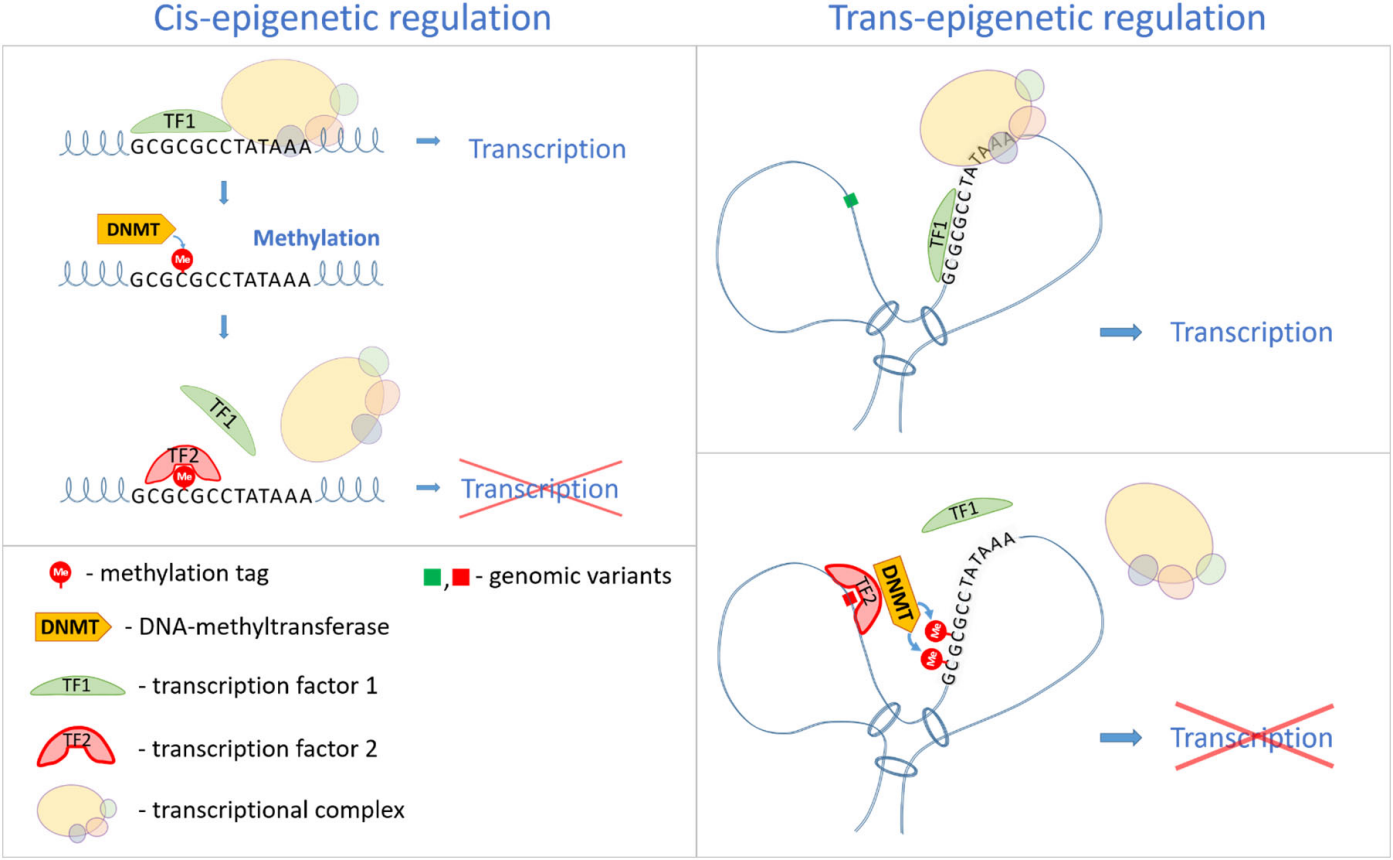
A scheme of possible mechanisms of the influence of genetic variants located either in the cis- or trans- position relative to the CpG-site on the level of methylation of this CpG-site and gene transcription.

Genetic variants specific to a particular individual can determine the level of methylation of particular CpG-sites, located either in the cis- (near the site, within ~1 Mbit/s) or in the trans- position (at a greater distance or even on a different chromosome) relative to this genetic variant (145) (**Figure 3**). Single nucleotide substitutions in target sites can disrupt transcription factor interaction with DNA, altering CpG-site accessibility for DNMTs, leading to changes in methylation patterns and, consequently, gene expression levels. Considering the three-dimensional organization of the genome, changes in nucleotide sequence can influence not only nearby sites but also distant genomic regions (145). This has been confirmed experimentally. Gibbs et al., using 600 brain tissue samples, demonstrated statistically significant correlations between genetic variability, gene expression, and DNA methylation, especially when the SNP was close to the methylation site (146). Bell et al., analyzing associations with over three million SNPs, identified 180 CpG sites in 173 genes associated with nearby genetic variants, confirming that SNPs can influence both DNA methylation and gene expression levels (147).

It is important to note that DNA methylation profiles, along with dependence on environmental influences, have a clear genetic basis. This is supported by studies investigating variability in DNA methylation patterns between different tissues of the same individual. A study conducted by Davis and co-authors demonstrated that the inter−tissue differences in DNA methylation in one person significantly exceeds the interindividual differences within one tissue, while the “pattern” of inter−tissue differences in DNA methylation, for example, between the brain and blood in different individuals is similar. (148). This implies that interindividual methylation differences are at least in part determined by a genetic background, for example, in the form of single nucleotide polymorphisms that arise in germline cells and are common to all tissues of a person. Furthermore, there is evidence suggesting that genetic variants themselves may underlie the transgenerational inheritance of DNA methylation patterns (145, 149).

Pathogenic or potentially pathogenic genetic variants, as mentioned, can also impair the properties of effector proteins. In the context of mental illnesses, these could include neurotransmitter receptors, voltage-gated channels, enzymes for neurotransmitter synthesis/inactivation/uptake, neurotrophic factors and their receptors, guidance molecules and their receptors, etc. (52, 150). However, in this review, we will limit ourselves to considering the possibility of the influence of genetic variants in genes that determine interaction with the external environment and the transmission of potentially stressful signals.

To date, a number of key genetic variants have been identified that manifest primarily when interacting with an adverse emotional environment. Research focus has centered on polymorphisms in genes associated with neurotransmitter systems and the stress response, such as *BDNF*, *NR3C1*, *DRD2/4*, *COMT*, *MAOA*, and *SLC6A4* (86, 151, 152).

A classic example is the *SLC6A4* gene, encoding the serotonin transporter. A polymorphism in its promoter region leads to a short (S) or long (L) allele. Carriers of the short S allele who experienced prenatal psychosocial stress subsequently more frequently demonstrate negative affectivity and increased susceptibility to affective disorders. In contrast, carriers of the long L allele, which ensures higher gene transcription, proved to be more resistant to negative environmental influences (153–155).

One of the most deeply studied examples of gene-environment interaction is the rs1360780 polymorphism in the *FKBP5* gene (156). Klengel and the co-authors investigated the functional significance of the rs1360780 polymorphism within the *FKBP5* gene, and found that this polymorphism reveals itself on the background of childhood-experienced stress. The rs1360780 variant is located in the functional region of the enhancer at a distance of 488 bp from GRE within the intron 2 and can represent either a stable C/G allele or an A/T risk allele, forming an additional TATA box. This attracts the TATA-binding protein (TBP) to the newly formed transcription start site producing the shortened FKBP51 protein, as TBP effectively recognizes the rs1360780-A variant. At the very same time, *FKBP5* gene expression is regulated by three-dimensional interactions of the intron 7 of the *FKBP5* gene with its own transcription start site (TSS), enhancing FKBP51 protein production. And the rs1360780-A variant turned out to coincide with increased demethylation of CpG sites near and within the GRE in the intron 7 of *FKBP5* gene (in childhood-stressed individuals), that leads to the activation of *FKBP5* expression (157). This demonstrates the complexity of gene expression regulation and how it can be dramatically changed by a single but significant genomic variant. Thus, the childhood stress overlapping a certain genomic variant causes sustained increased expression of *FKBP5*, disrupts negative feedback to the HPA axis, increasing the risk of manifestation of mental disorders.

Another important gene involved in transmitting environmental signals is the *MECP2* gene. Multiple mutations in this gene are known to cause Rett syndrome, characterized by severe neurological and cognitive impairments. Since the MeCP2 protein is a key reader of methyl marks and regulator of transcription for numerous genes critical for brain development and stress response, even less numerous and malignant variants can significantly increase the risk of mental disorders by modulating the epigenetic landscape in response to stress stimuli (158–160).

It should be noted that results from studies on the effect of specific gene-environment interactions can be quite contradictory. This may be explained by differences in assessing environmental exposures, different age periods of analysis, and also by the phenomenon of differential stress susceptibility. According to this concept, individuals who are most vulnerable to the negative effects of the environment can simultaneously benefit the most from relatively favorable conditions, such as the simple absence of adverse factors. A striking example is the rs6265 polymorphism (Val66Met) in the *BDNF* gene. Some studies show that carriers of the Met allele exhibit more depressive symptoms in favorable conditions, but in an unfavorable environment their results may be better than those of carriers of the Val allele. Other works indicate only the negative effect of this option when experiencing difficult life situations (161, 162).

Therefore, an individual’s genetic pattern functions as a moderator, shaping a unique stress sensitivity profile that determines personal risk trajectory following environmental challenges (50). The accumulation of such data deepens our understanding of the etiology and pathogenesis of mental and cognitive disorders and opens broad diagnostic, preventive, and therapeutic possibilities. Obtaining this data is a slow process, as it requires experimental validation of identified or predicted genetic variants using cellular and animal models (163–165). Given the complexity of molecular interactions, the range of their participants, possible partners, and binding sites can be quite extensive. This suggests the existence of a large number of potentially pathogenic genetic variants. Currently, we only know a few of them; we are at the beginning of a long journey.

## 6 Conclusion

The analysis of accumulated data convincingly demonstrates that stress-induced changes in the epigenome represent a key molecular bridge connecting exposure to adverse environmental factors with an increased risk of the onset of mental disorders. As summarized in the article, severe or chronic stress, especially during critical periods of CNS development, initiates a cascade of events through the activation of hormonal axes (primarily the HPA axis) and neurotransmitter systems, altering the activity of epigenetic modifiers (DNMT, TET, HDAC, KMT). This leads to persistent changes in DNA methylation patterns and histone modifications in the regulatory regions of genes that are critical for neurogenesis, synaptic plasticity, and stress response (such as *BDNF*, *NR3C1*, *FKBP5*, *SLC6A4*, *RELN*, *MECP2*, among others). These epigenetic shifts disrupt the normal program of brain development and function, which, combined with an individual’s genetic background, may lay the material basis for the pathogenesis of a mental disease.

This research direction is highly promising, as it can elucidate the underlying mechanisms of psychiatric disorders and create new opportunities for diagnosis and therapy. However, translating these findings into diagnostic tools requires more systematic and comprehensive studies. And the primary objective is to identify and validate stable epigenetic signatures (episignatures) in easily accessible tissues, such as blood or saliva, that reliably reflect pathological processes ongoing in the CNS. These episignatures could serve as non-invasive biomarkers for assessing the risk of development or early diagnosis of mental disorders. However, this field requires the standardization of approaches to overcome existing data fragmentation and ensure the reliability and accuracy of such methods.

Understanding the molecular mechanisms of stress-induced epigenetic reprogramming opens possibilities for developing targeted therapy. The most promising approach seems to be the development of drugs that selectively correct the activity of specific epigenetic modifiers (e.g., inhibitors of specific HDACs or DNMTs), and potentially guide them to the target genomic region for introducing the required epigenetic modification. The ultimate goal is to create a solid foundation for personalized epigenetic therapy, allowing for the correction of deviations considering the unique genetic-epigenetic profile of the patient. We believe that this could fundamentally change approaches to the treatment and prevention of mental disorders in the future.
